# Long-Term Non-invasive Ventilation in Children With Down Syndrome: A Systematic Review

**DOI:** 10.3389/fped.2022.886727

**Published:** 2022-05-23

**Authors:** Summer Hudson, Tamer Abusido, Meghan Sebastianski, Maria L. Castro-Codesal, Melanie Lewis, Joanna E. MacLean

**Affiliations:** ^1^MD Program, Department of Pediatrics, Faculty of Medicine & Dentistry, University of Alberta, Edmonton, AB, Canada; ^2^Divisions of Respiratory Medicine, Department of Pediatrics, Faculty of Medicine & Dentistry, University of Alberta, Edmonton, AB, Canada; ^3^Pediatric Pulmonology Division, Pediatric Department, King Abdullah Specialized Children's Hospital, Ministry of National Guard Hospital Affairs, Riyadh, Saudi Arabia; ^4^Alberta Strategy for Patient Oriented Research (SPOR) Support Unit, Alberta Research Centre for Health Evidence, University of Alberta, Edmonton, AB, Canada; ^5^Division of General & Community Pediatrics, Department of Pediatrics, Faculty of Medicine & Dentistry, University of Alberta, Edmonton, AB, Canada; ^6^Women's & Children's Health Research Institute, Faculty of Medicine & Dentistry, University of Alberta, Edmonton, AB, Canada

**Keywords:** pediatric, obstructive sleep apnea, Trisomy 21, continuous positive airway pressure, bilevel positive airway pressure

## Abstract

**Context:**

Children with Down syndrome are at risk for obstructive sleep apnea, which may not be resolved by adenotonsillecotmy, as well as other respiratory disorders that may impact breathing during sleep. Long-term non-invasive ventilation, including continuous and bilevel positive airway pressure delivery, is an alternate treatment strategy.

**Objective:**

To assess the use and outcomes of long-term non-invasive ventilation in children with Down syndrome including comparison to other children using long-term non-invasive ventilation.

**Data Sources:**

The search strategy for the scoping review used Medical Subject Headings (MeSH) and free-text terms for “child” and “non-invasive ventilation.” MEDLINE (Ovid), Embase (Ovid), CINAHL (Ebsco), Cochrane Library (Wiley), and PubMed databases were searched (1990-2021).

**Study Selection:**

The scoping review results were searched to identify studies including data on at least three children with Down Syndrome using long-term non-invasive ventilation.

**Data Extraction:**

Study characteristics, subject characteristics, technology type, and outcome measurements were extracted.

**Results:**

A total of 28 articles included 543 children with Down syndrome using long-term non-invasive ventilation. Children with Down syndrome accounted for 18% of children using long-term non-invasive ventilation. Data on efficacy, feasibility, and adherence in children with Down syndrome are comparable to other children. Children with Down syndrome may have greater difficulty initiating long-term non-invasive ventilation, longer time to establish use, and a higher rate of inability to establish use. Outcome data is limited but suggest favorable impact on cardiac function and attention.

**Limitations:**

Articles related to long-term non-invasive ventilation use in adolescents and young adults may have been excluded.

**Conclusions:**

Children with Down syndrome make up a significant portion of the population of children using long-term non-invasive ventilation. While there is more limited data available with respect to the use and outcomes for children with Down syndrome compared to the other children, long-term non-invasive ventilation is an effective and well-tolerated therapy with no clear differences in the use or outcomes for children with Down syndrome. Additional work is needed to understand potential challenges around establishing long-term non-invasive ventilation use in children with Down syndrome.

**Systematic Review Registration:**

https://www.crd.york.ac.uk/prospero/display_record.php?RecordID=206533, identifier: CRD206533.

## Introduction

Down syndrome (DS), or Trisomy 21, is the most common chromosomal disorder, with an estimated prevalence of 14 per 10,000 live births in the United States (1). DS is associated with several anatomical features that predispose children to the development of obstructive sleep apnea (OSA), including macroglossia, midfacial and mandibular hypoplasia, muscle hypotonia, adenotonsillar hypertrophy, and subglottic or tracheal stenosis (2). These features are often compounded by the presence of obesity, hypothyroidism, and gastro-esophageal reflux, all of which are common comorbidities of DS (2). As a result, OSA occurs with a heightened prevalence of 34–76% in this population, in contrast to the 1–4% prevalence observed in typically developing children (3, 4). In addition to a higher prevalence of OSA, children with DS have a high rate (24–48%) of residual OSA after adenotonsillectomy as first line treatment (5–7). DS also confers susceptibility to other respiratory conditions that may impact breathing during sleep including recurrent upper respiratory tract infections, airway malacia, tracheal bronchus, and pulmonary hypertension (8, 9). Children with DS and residual OSA after surgery, contraindications to surgery, or other respiratory conditions that impact breathing during sleep are considered for treatment with non-invasive ventilation (NIV), including continuous and bilevel positive airway pressure (CPAP, BPAP) delivery.

Long-term NIV (LT-NIV), defined as respiratory support administered through an interface outside the airway, has become a mainstay of treatment for OSA and other types of sleep-related breathing disorders as well as respiratory insufficiency or failure in children. An increase in the use of NIV is likely attributable to advancements in the technology available, a positive attitude shift toward home-care, and increasing acceptance of NIV as a viable therapeutic option (10). Given the high prevalence of OSA and other sleep-related breathing disorders among children with DS, use of LT-NIV in this population is common. While there is considerable literature reporting on the use of LT-NIV in the broader pediatric population (10) work specific to its use in children with DS is more limited. Specific anatomical susceptibilities, as well as cognitive and behavioral challenges may complicate the use and alter anticipated outcomes of LT-NIV in children with DS. A better understanding of the benefits and challenges of LT-NIV use for children with DS is important to aid clinicians and families with decision making as well as informing health policy around funding for equipment and support for children and families using LT-NIV.

The aim of this systematic review is to assess the use and outcomes of LT-NIV in children with DS and to determine similarities and differences in its use when compared to other children using LT-NIV. Our research question was: Does LT-NIV use differ for children with DS compared to other children with respect to conditions it is used to treat, adherence to LT-NIV treatment, or anticipated outcomes of this therapy?

## Methodology

### Protocol and Registration

This review was conducted in accordance with the guidelines of the Preferred Reporting Items for Systematic Review and Meta-Analyses (PRISMA) (11). The protocol was documented and registered in the PROSPERO database (CRD42020206533).

### Eligibility Criteria

The inclusion criteria for this systematic review were as follows: (i) DS; (ii) age 0-18 years; and (iii) LT-NIV use, defined as respiratory support delivered *via* an interface outside the airway for at least 3 months in a non-acute care setting. Studies reporting on a broader range of conditions were included only if they provided separate data on subjects with DS. If articles included subjects over 18 years of age, the mean age at NIV initiation needed to be under 18 years for inclusion. There were no restrictions on study design, outcome eligibility, or language at the title/abstract stage; language was limited to English, French, Spanish, Catalan, and Portuguese at the full text stage. To be included, studies had to report on a minimum of three children with DS.

### Information Sources and Search

This systematic review is an extension of a scoping review on LT-NIV in children (10). The search strategy for the scoping review, developed for MEDLINE (Ovid) and later translated to additional databases, used Medical Subject Headings (MeSH) and free-text terms for “child” and “non-invasive ventilation” (Supplement Table 1). Since the first study of LT-NIV use in children was published in 1992, studies of humans published from 1990 onwards were searched in the following databases: Ovid MEDLINE, Embase (Ovid), CINAHL (Ebsco), Cochrane Library (Wiley; Inception to Present), and PubMed. The original search was conducted between November 17-28, 2014 and updated most recently on March 25, 2021.

### Study Selection

The titles and abstracts of articles identified by the literature search were reviewed for eligibility for full text retrieval by two reviewers (MCC, TA, JEM). Studies in English, French, Spanish, and Portuguese were considered eligible for full-text retrieval and were reviewed by two reviewers (MCC, TA, JEM). The final list of studies eligible for inclusion from the scoping review were then full-text reviewed by two reviewers (SH and JEM) to identify studies that met the inclusion criteria for this systematic review. Any disagreement at the screening, eligibility, or inclusion levels were discussed by the reviewers until a consensus was established.

### Data Extraction and Items

Data were collected and entered into a pre-established form in Microsoft Excel (version 16.38, Microsoft Corporation, 2020). These data items included author's name, year of publication, country of publication, journal, study design, study duration, sample size, age at NIV initiation, proportion of females, NIV type, and outcomes. One reviewer (SH) extracted the data and a second reviewer (JEM) verified the data extraction.

### Risk of Bias and Quality Assessment

Independent assessment for risk of bias and quality assessment of each article was conducted by two reviewers (SH, JEM) using the Cochrane Risk of Bias in Non-Randomized Studies of Interventions (ROBINS-I) tool (12) and the Grading of Recommendations Assessment, Development and Evaluation tool (GRADE) (13). Disagreements were resolved through discussion and consensus.

### Synthesis of Results

Studies were grouped by DS subject type; studies that included children with DS as part of a broader cohort of children using LT-NIV and studies that exclusively included children with DS using LT-NIV. Numeric data [median, 95% confidence interval (CI)] was summarized where available and examined for meta-analysis with the data summarized narratively otherwise. Where additional analysis to compare children with and without DS within an article was possible, *p* < 0.05 denoted significant differences between these groups.

## Results

### Study Selection and Characteristics

**Figure 1:**
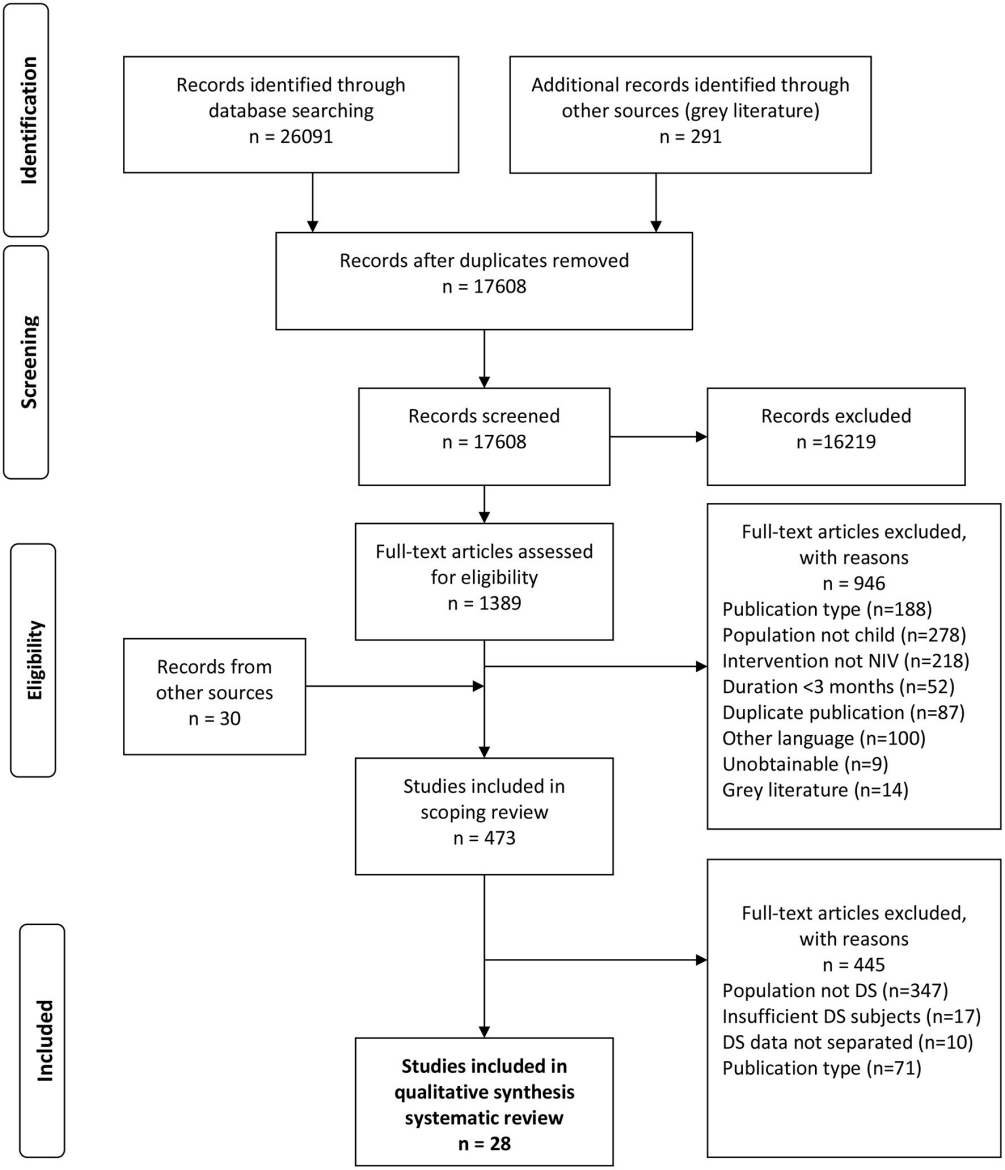
Flow diagram outlining the study selection process for the systematic review, following the Preferred Reporting Items for Systematic Reviews and Meta-Analyses (PRISMA) guidelines (11). The protocol and scoping review results provide the details of the search strategy (10).

Following removal of duplicates and inclusion of additional records, the scoping review search strategy identified 17608 unique records for review (Figure 1). After screening titles and abstracts, 1389 records were eligible for full-text review and 473 articles met inclusion criteria for the scoping review on LT-NIV use in children. Final full-text review identified 28 articles meeting the inclusion criteria for this systematic review. These studies included 543 children with DS who used LT-NIV. Publication dates ranged from 1995 to 2021. Articles originated from nine countries with the majority of studies stemming from North America (16/28, 57%) and Europe (9/28, 32%).

Most studies were retrospective (21/28, 75%), and single-center (25/28, 89%), and all were quantitative. The majority of studies were observational (26/28, 93%) and described as cohort (7/28, 25%), or cross-sectional (19/28, 68%) studies. Two studies (7%) were randomized trials. Across the 28 studies, 20 included children with DS in a broader group of children (Table 1) while eight were exclusively on children with DS (Table 2). Data for quantitative synthesis was limited to age at NIV initiation and proportion of female subjects with the remainder of the data summarized using narrative synthesis.

**Table 1:** Summary of studies including data on children with Down syndrome within a larger group of children using long-term non-invasive ventilation.

**References**	**Pro/ Retro**	**Study design**	**Study duration**	**Indications for use**	**Total on NIV**	**Total %F**	**Age overall [mean ±SD, (range)]**	**T21 on NIV (*n*, % DS in cohort)**	**NIV mode (# CPAP/BPAP)**	**Summary of data relevant to children with DS**
Guilleminault et al. (14)	P	Ob (CS)	5 mos-12 years follow-up	OSA	74	53	24 ± 9 wk	7 (9%)	CPAP	86% (6/7) of infants with DS discontinued CPAP between 4-7 years after airway surgery [compared to 34% (22/65) of non-DS]; 43% (3/7) discontinued because of improvements on PSG. [numbers for PSG do not add up for non-DS]
O'Donnell et al. (15)	R	Ob (CS)	October 1999–July 2003	OSA (AHI>1 events/h; if AHI 1-5 events/h, associated symptoms), post-AT or poor candidates for AT	79	33	10 ± 5.1 y	22 (30%)	CPAP	Of the children started on CPAP without AT, 19% (7/22) were children with DS [compared to 52% (30/57) of non-DS]. 50% (11/22) of children with DS used a full face mask [compared to 7% (4/57) of non-DS] and 27% (6/22) were referred to psychology for assistance [compared to 14% (8/57) of non-DS]. Compliance did not differ between those with and without impaired cognition, with and without AT, full face vs. nasal mask, with and without psychological support [no data specific to DS]. 20% of children took longer than 90 d to use CPAP; 60% of these were children with DS
Girbal et al. (16)	R	Ob (CS)	January 2017–March 2012	OSA (AHI ≥ 1), OSA + hypoventilation (median TcCO2 > 45 mmHg or morning pCO2 > 45 mmHg)	68	41	6.58 y (15–171 mos)	5 (7%)	CPAP/BPAP (3/2)	Children with DS started NIV at 10 years (IQR 3.8-15) [cf 6.6 (IQR 1.25-14.2) of full group]. All children used nasal mask or prongs. Of 16 started on BPAP, 12% (2/16) were children with DS; of children with DS 40% (2/5) were on BPAP compared to 22% (14/63) of non-DS
Amaddeo et al. (17)	R	Ob (CS)	October 2013–September 2014	OSA, alveolar hypoventilation alone or associated with OSA. PG/PSG criteria (mean 4 of 6): Min SpO2 <90%, Max TcCO2 > 50 mmHg, time with SpO2 <90% ≥ 2%, time spent with TcCO2 > 50 mmHg ≥ 2%, ODI >1.4 events/h, AHI >10 events/h	76	49	Acute: 0.3 y (0.1–13.5 y) Sub-acute: 0.6 y (0.2–18.2 y) Chronic: 1.6 y (0.1–19.5 y)	6 (8%)	CPAP/BPAP	Of six children with DS starting on NIV, 17% (1/6) were started during a hospitalization without PG/PSG and 83% (5/6) were started after PG/PSG [compared with non-DS: 21% (15/70) started in PICU, 24% (17/70) started during a hospitalization without PG/PSG, 54% (38/70) started after PG/PSG]
Amaddeo et al. (18)	P	Ob (cohort)	March 2015–January 2017	OSA (>5 events/h) despite optimal surgical and/or medical treatment	31	39	8.9 (0.8-17.5) y	7 (23%)	CPAP	Of four patients who never achieved CPAP use ≥4 h, 75% were adolescents with DS [DS 43% (3/7) vs. non-DS 4% (1/24)]. One of these children had a second adenoidectomy that resolved OSA and two were switched to high flow air by nasal cannula but did not comply with
										this. One with AHI 9 events/h did not return for follow-up and the other underwent orthodontic treatment
Chong et al. (19)	R	Ob (CS)	January 2009–June 2018	OSA by polysomnography	198	28	13.1 ± 3.6 y	23 (12%)	CPAP	12% of DS subjects underwent AT prior to NIV initiation (compared to 67% overall). Distribution of optimal CPAP in the DS subgroup did not vary by OSA severity (compared to overall variation in optimal CPAP by OSA severity). Multivariable model included DS as a predictor of optimal CPAP; overall, the model explained 31% of the variance in optimal CPAP pressure
Griffon et al. (20)	R	Ob (cohort)	January 2017–March 2018	NR	79	41	6 (IQR 1.5-1.4) y	13 (16%)	CPAP/NIV/ HFNC	Two of 13 (15%) subjects with DS had abnormal overnight gas exchange. Both had mean CO2 > 50 mmHg and AHI >25 events/h; one switched from CPAP to HFNC because of non-compliance with CPAP with follow-up hospital recording showing no hypoventilation and the other had an increase in CPAP with persistence of hypoventilation on follow-up hospital recording
MacDonagh et al. (21)	R	Ob (cohort) with com- parison to non-DS children using LT-NIV	March 2014–August 2019	OSA by polysomnography	DS 44, Non-DS 62	DS 55%, Non-DS 55%	DS 4.76 ± 7.92 y, Non-DS 5.18 ± 5.64	44 (41%)	NIV (only CPAP mentioned)	Adherence to NIV in children with DS is satisfactory compared to non-DS children. Children with DS with known congenital cardiac disease who underwent cardiac surgery had lower adherence compared to DS children without a history of cardiac surgery

*AT, adenotonsillectomy; BMI, body mass index; BPAP, bilevel positive airway pressure; DS, down syndrome; CPAP, continuous positive airway pressure; CS, cross-sectional; HFNC, high flow nasal cannula; IQR, interquartile range; mmHg, milometers of mercury; mo, month; NIV, non-invasive ventilation; Ob, observational; OSA, obstructive sleep apnea; P, prospective; R, retrospective; SD, standard deviation; wk, week; y, year*.

**Table 2:** Summary of studies exclusively on children with Down syndrome using long-term non-invasive ventilation.

**References**	**Study design**	**Center**	**Study duration (y)**	**Total DS (N)**	**%Female**	**Age overall [y; mean ±SD, median (IQR), (range)]**	**BMI/Obesity**	**DS on NIV (n)**	**Indications for Use**	**NIV mode (#CPAP/BPAP)**	**Additional data**
Rosen (22)	R, Ob (cohort)	Single	5.5	29	NR	<2 y	NR	6	OSA	CPAP	50% of infants with DS treated with CPAP outgrew OSA
Shete et al. (23)	R, Ob (cohort)	Single	8	11	36	8.5 y	NR	6	Residual OSA post-AT	CPAP/BPAP	A high proportion of children with DS and OSA will require treatment with NIV after AT (55%)
Brooks et al. (24)	P, Ob (cohort)	Single	1.08	25	44	10.2 ± 3.9 y	NR	7	OSA prefer non-surgical, residual OSA post-AT	CPAP	At baseline, 40% of those tested had OSA with no difference in neurocognitive testing in those with and without OSA. CPAP was successful in 43% [3/7; compared with 66% (2/3) successful AT]. Those who were successfully treated showed greater improvement in attention [data not presented separately for NIV]
Esbensen et al. (25)	R, Ob (cohort)	Single	4	954	45	12.6 ± 5.4 y (5–21 y)	525 (55%) overweight	66	OSA	PAP	At baseline, 36% of those tested had OSA (only 48% underwent PSG). The only factor that predicted PAP use was the presence of OSA. Gender, age, race, BMI, behavioral sleep disorder were also included as predictors
Konstantinopoulou et al. (26)	P, Ex (DB RCT)	Single	0.33	23	39	10 y (IQR 9.0–14.3)	BMI Z-Score: 1.4 (IQR 0.9–2.2)	20	OSA, residual OSA post-AT, excl unrepaired major CHD and chronic lung disease (except well-controlled asthma)	CPAP	At baseline, 87% had OSA and no child showed evidence of pulmonary hypertension. While there was no significant differences in cardiovascular outcomes between those randomized to actual vs. sham CPAP, CPAP use correlated with improvement in left ventricular dysfunction
Dudoignon et al. (27)	R, Ob (cohort)	Single	2.5 (mean treatment duration 2 ± 1 y)	57	46	Overall: 6.2 ± 5.9 y, NIV: 5.9 ± 4.9 y; No NIV: 6.9 ± 7.7 y	BMI: 19.0 ± 4.9; BMI: z-Score: 1.7 ± 4.0	19	OSA prefer non-surgical, residual OSA post-AT, persistent alveolar hypoventilation despite CPAP	CPAP/BPAP (15/4)	33% of the cohort required NIV. Those who were treated with NIV did not differ from those who were not with regard to age and BMI but had higher mean AHI, OAI, ODI. NIV resulted in improvement in min SpO2, % time with SpO2 <90%, and ODI—while the pattern was the same for upper airway surgery, the difference from baseline was not statistically different. 16% (3/19) could be weaned from NIV. 26% (5/19) failed use of NIV
Trucco et al. (28)	R, Ob (cohort)	Single	5.83	60	38	5.7 y (3.1–9.4)	BMI 16.7 (IQR 14.6–18.3); BMI z-score: 0.89 (IQR −0.23–1.62)	25	OSA, OSA with raised overnight CO_2_	CPAP/BPAP (18/7)	At baseline, 45% had OSA, 32% nocturnal hypoventilation, 27% PHtn. 75% who were referred post-AT required NIV. 25% of those without prior AT started on NIV. 52% of children with pulmonary hypertension used NIV compared to 41% of those without. NIV pressures did not differ significantly at 2 years from those at establishment. 8% stopped NIV because of improvement and 8% switched form CPAP to BPAP with an improvement in adherence. Adherence did not differ for CPAP vs. BPAP at 4 mos or 1.9 y
Diskin et al. (29)	P, Ob (CS)	Multi	NR	393	44	7 y (4 mos-18 y)	NR	37	OSA	CPAP	Of 37 children started on CPAP, 44% were always or nearly always compliant with 59% reporting CPAP as very or extremely beneficial. Nine children (24%) reported never using CPAP with 55% of these reporting no benefit

*AT, adenotonsillectomy; BMI, body mass index; BPAP, bilevel positive airway pressure; CHD, congenital heart disease; DS, Down syndrome; CPAP, continuous positive airway pressure; CS, cross-sectional; Ex (DB RCT), experimental (double blink randomized controlled trial); IQR, interquartile range; mos, months; NIV, non-invasive ventilation; NR, not reported; Ob, observational; OSA, obstructive sleep apnea; PAP, positive airway pressure; P, prospective; R, retrospective; SD, standard deviation; y, year*.

### Use of Long-Term NIV in Children With DS Compared to Other Children

In the 20 studies that included children with and without DS using LT-NIV, children with DS made up 18% (357/2005) of the population (Table 1). The proportion of children with DS using LT-NIV did vary widely across studies (7–44%). The median age of subjects in studies including children with and without DS, which included children with OSA who received treatments other than NIV, overlapped with those in studies focused on DS [9.0 (95% CI 6.0–11.3) vs. 8.5 (95% CI 6.2–10.9) years]. The proportion of female subjects in the broader studies is also similar to those studies focused on DS [39% (95% CI 35–44) vs. 44% (33–47%)]. Twelve of these studies included only the number of children with DS using LT-NIV so were not analyzed further (30–41).

Of the remaining eight studies that included children as part of a broader group of children using LT-NIV (Table 1) (14–21), only one provided direct comparisons between children with DS and non-DS children (21). In this study, children with DS made up 41% (44/106) of children using LT-NIV with overall similar age and sex distribution between the DS and non-DS groups; in the sub-group of children 0-5 years of age, the DS group was younger than the non-DS group [0.42 (interquartile range, IQR, 1.04) vs. 2.1 (IQR 3.4) years]. The apnea-hypopnea index (AHI), as a measure of OSA severity, was higher in the DS group (DS: 16.8 ± 1.85 vs. non-DS: 11.73 ± 1.5 events/h). While LT-NIV adherence was greater in the DS group (DS: 79% vs. non-DS: 72% of days used) there was no difference in adequate usage (>4 h) on the nights used. Data from machine downloads showed that AHI (5.71 ± 0.47 vs. 4.42 ± 0.27 events/h), system leak, and percentage time in excess leak from device downloads were higher in the DS compared to non-DS group though there were significant improvements in AHI in both groups with LT-NIV use.

The other seven studies reporting on children with DS as part of a broader group of children using LT-NIV included 83 children with DS (Table 1) (14–20). The indications for LT-NIV was reported for six of these studies; while OSA was an indication for all six, two studies included hypoventilation without OSA (16) one study limited indications to residual OSA post-adenotonsillectomy or medical management (18), and one limited indications to OSA post-adenotonsillectomy or preference for non-surgical management/poor surgical candidate (15). Four of these studies included only CPAP treatment (14, 15, 18, 19) while two included CPAP/BPAP (16, 17), and one included CPAP/BPAP as well as high flow nasal cannula (20).

Age of LT-NIV initiation for children with DS was reported in one study and was 10 y (IQR 11.2) compared to 6.6 (IQR 12.95) for the full group (16). One study reported the proportion of children started on CPAP without prior adenotonsillectomy or adenoidectomy; 32% (7/22) were children with DS compared to 53% (30/57) of non-DS children (Chi-square 2.8, *p* = ns) (15). A single study reported on location of NIV initiation and showed that children with DS were started during a hospitalization (17%, 1/6) or after polygraphy/polysomnography (83%, 5/6) while non-DS children started in the PICU (21%, 15/70), during a hospitalization (24%, 17/70), and after polygraphy/polysomnography (54%, 38/70) (17). Interface type for children with DS was reported in two studies. One reported the use of full face mask in 50% (11/22) of children with DS in contrast to 7% (4/57) of non-DS children (Chi-square 19.1, *p* < 0.001) (15). The second included five children with DS and reported that all children used a nasal mask or prongs (16). This study also reported on NIV mode where 60% (3/5) of children with DS were started on CPAP and 40% (2/5) on BPAP compared to 78% (49/63) started on CPAP and 22% (14/63) on BPAP for non-DS children (Chi-square 0.84, *p* = ns).

Data on adherence in DS and non-DS children were reported in two studies. One study that included 22 children with DS (19% of subjects) reported that adherence in the full group did not differ between those with and without impaired cognition, with and without adenotonsillectomy prior to NIV, full face vs. nasal mask, and with and without psychological support for NIV initiation (15). This same study reported that 27% (6/22) of children with DS were referred for psychological assistance compared to 14% (8/57) of non-DS children (Chi-square 1.91, *p* = ns). Finally, while 20% of children took longer than 90 days to establish CPAP use, 60% (6/14) of this group were children with DS (Chi-square 1.91, *p* = ns). In the second study which included 31 children, four never initiated CPAP use ≥4 h; the rate of never initiating CPAP use ≥4 h was 43% (3/7) for children with DS compared to 4% (1/24) for non-DS children (Chi-square 6.9, *p* < 0.01) (18).

### Use of Long-Term NIV in Children With DS

The eight studies focused on DS included 186 children who were using LT-NIV (Table 2). Four studies included data on body composition with 55% of all subjects being obese in one study (25) and three studies reporting BMI z-scores of 1.4 (IQR 1.4) in subjects with OSA in one cohort, 1.7 ± 4.0 for the full cohort in the second (some of whom were treated with surgery rather than NIV), and 0.89 (IQR 1.85) in the third (26–28). The indications for use were similar to the studies reporting data on children with DS in larger cohorts of children using LT-NIV; OSA, OSA post-adenotonsillectomy, OSA with preference for non-surgical management, and hypoventilation or raised CO_2_ overnight. One study reported that 55% of children with DS and OSA required LT-NIV after adenotonsillectomy (23) where another reported that 33% of their DS cohort required LT-NIV after baseline sleep study (27).

Interface type was specified in two studies with 75% (33/44) of children using nasal masks and 25% (11/44) using nasobuccal or full face masks (27, 28). Subjects in four studies used exclusively CPAP (22, 24, 26, 29) while the remaining four studies reported on CPAP and BPAP or PAP usage (23, 25, 27, 28). Two studies reported the proportion of subjects using CPAP and BPAP; overall, 75% (33/44) used CPAP and 25% (11/44) used BPAP (27, 28). Two studies reported on NIV pressure settings and adherence (27, 28) with only one reporting pressure settings at follow-up (28). CPAP pressures ranged from 6–10 cmH_2_O with BPAP median inspiratory positive airway pressure of 12 (range 12–17) and 14 (IQR 6) cmH_2_O, and median expiratory positive airway pressure of 8 (range 5–8) and 7 (IQR 4) cmH_2_O (27, 28). The study that included pressure settings at follow-up showed no change in CPAP or BPAP pressures after a median follow-up of 1.9 y (28). With respect to adherence, one study reported an average use of 8 h 46 ± 6 h 59 per night and 82% of children showing use >4 h/night (27) while another reported 56% regular use at four months and 46% at 1.9 y with a median use/night of 4 h (IQR 6) for CPAP and 8 h (IQR 3) for BPAP at 1.9 y (28), and a third reported average nightly use of 116 min (IQR 69) (26). Adherence was described as high in the first study, good with no difference between CPAP and BPAP in the second, and low though typical of other studies in the third. In a study of 37 children initiated on CPAP, 44% were rated by parents as “always” or “nearly always adherent” while 24% were “never” adherent; children who were adherent were more likely to report perceived benefit (29).

Three studies reported on outcomes of NIV treatment in children with DS. One study compared treatment outcomes between LT-NIV and upper airway surgery (adenoidectomy ± tonsillectomy ± turbinectomy) and reported improvements, compared to baseline, in oxygen parameters on sleep studies for children using LT-NIV; while the same pattern of changes was seen after surgery, these differences did not differ from baseline measures (27). Of note, the criteria for starting LT-NIV differed from the criteria for surgery (AHI>10 vs. >5 events/h) so the groups were not comparable at baseline (27). In a randomized trial of CPAP vs. sham CPAP for OSA in children with DS, where none of the children had pulmonary hypertension at baseline based on echocardiography, cardiovascular outcomes did not differ between groups at 4-months follow-up though there was an improvement in LV diastolic function with CPAP use (26). In a study of sleep, sleep apnea, and neuropsychological function in children with DS, those successfully treated for OSA, either with adenotonsillectomy or CPAP, showed greater improvement in attention though results were not presented separately by treatment type (24). Finally, LT-NIV was ceased because of improvements in 8–50% of children with DS across three studies (22, 27, 28).

### Risk of Bias and Quality Assessment of Outcomes

Overall risk of bias across studies was serious for all studies (Table 3). This was predominantly attributable to study design as most were observational. The included data was heterogenous in nature with limited comparative evidence; quality assessment tools could not be applied.

**Table 3:** Assessment of risk of bias in individual studies included in the systematic review of long-term non-invasive ventilation use in children with down syndrome using the risk of bias in non-randomized studies—of interventions (ROBINS-I) tool.

**References**	**Confounding**	**Selection**	**Measurement of intervention**	**Missing data**	**Measurement of outcomes**	**Selection of reported results**	**Overall risk of bias (RoB) assessment**
**Include data on down syndrome and non-down syndrome children**							
Guilleminault et al. (14)	Serious	Serious	Serious	Serious	Serious	Moderate	Serious
O'Donnell et al. (15)	Serious	Moderate	Moderate	Serious	Moderate	Moderate	Serious
Girbal et al. (16)	Serious	Serious	Moderate	Serious	Serious	Serious	Serious
Amaddeo et al. (17)	Serious	Serious	Serious	Serious	Serious	Serious	Serious
Amaddeo et al. (18)	Serious	Moderate	Moderate	Serious	Moderate	Serious	Serious
Chong, et al. (19)	Serious	Serious	Serious	Moderate	Moderate	Serious	Serious
Griffon et al. (20)	Serious	Serious	Serious	Moderate	Moderate	Moderate	Serious
MacDonagh et al. (21)	Serious	Serious	Serious	Moderate	Moderate	Serious	Serious
**Include data only on children with down syndrome**							
Rosen (22)	Moderate	Serious	Serious	Serious	Serious	Serious	Serious
Shete et al. (23)	Serious	Serious	Moderate	Serious	Serious	Serious	Serious
Brooks et al. (24)	Serious	Serious	Moderate	Serious	Serious	Serious	Serious
Esbensen et al. (25)	Serious	Serious	Moderate	Serious	Serious	Serious	Serious
Konstantinopoulou et al. (26)	Serious	Moderate	Serious	Serious	Serious	Moderate	Serious
Dudoignon et al. (27)	Serious	Serious	Serious	Serious	Serious	Moderate	Serious
Trucco et al. (28)	Serious	Serious	Moderate	Moderate	Serious	Serious	Serious
Diskin et al. (29)	Serious	Serious	Serious	Serious	Serious	Serious	Serious

*Studies that only report on the number of children with down syndrome using long-term non-invasive ventilation were excluded from risk of bias assessment*.

## Discussion

To our knowledge, this is the first systematic review on LT-NIV use in children with DS. The results identified limited data that could be combined for quantitative analysis and serious risk of bias across all included studies. Only one study provided direct comparison between children and without DS who were using LT-NIV. This is despite children with DS accounting for a high, though variable, proportion of children using LT-NIV across studies. While the limited amount of data synthesis and high risk of bias data precludes strong conclusions, the results of this systematic review provide a summary of the available data and highlights direction for future research. Overall, there are no clear differences in the use or outcomes of LT-NIV in children with and without DS. Adherence did not differ from other children using LT-NIV. Children with DS, however, may have a greater need for additional support around initiation, take more time to establish use, and have a higher rate of inability to establish LT-NIV use compared to other children. Compared to other children using LT-NIV, children with DS may have higher residual AHI and leak on LT-NIV. LT-NIV use for OSA in children with DS may have positive impacts on oxygenation, heart function, and attention. Children with DS and OSA may have improvements in OSA such that LT-NIV can be ceased.

Children with DS account for a large proportion of children using LT-NIV. Children with DS experience OSA at disproportionately higher rates compared to typically-developing children (34-76 vs. 1-4%, respectively) (3, 4). Children with DS are less likely to be cured of OSA with removal of adenotonsillar tissue so are more likely to require additional treatment for OSA which would include LT-NIV (15). In addition to a high OSA risk, children with DS are at risk for neurocognitive impairments (42) that may be compounded by OSA related sleep disruption. With these risks, it is not surprising that DS is one of the most common syndromes associated with LT-NIV use (15, 19, 20, 39). What may be surprising is the limited data specific to the use and outcomes of LT-NIV use in children with DS. It is, however, important to recognize that despite a large body of work related to LT-NIV use in all children, most of this data is descriptive with little data on outcomes overall (10). The results of this systematic review provide a summary of the available data for DS, suggest there are potential benefits for cardiovascular and neurocognitive function, and that efficacy and adherence do not differ for DS as compared to other children using LT-NIV. While additional work is needed to understand the specific outcomes of LT-NIV use, there is sufficient data to support its use as a treatment for OSA in children with DS.

Although only one study compared the use of LT-NIV in children with DS to non-DS children, there appear to be some clinical characteristics and technology factors that may be important to consider for children with DS. Children with DS may be more likely to be started on LT-NIV after a polysomnography compared to other children using LT-NIV. This may reflect recommendations from the American Academy of Pediatrics that all children with DS should be screened for OSA with polysomnography by age 4 (43). In fact, polysomnography screening for OSA in children with DS increased after these recommendations were released (44, 45). While overall age appears similar in DS and non-DS children starting on LT-NIV, a higher proportion of children with DS may start on LT-NIV as infants. This is likely because children with DS make up a larger portion of infants with OSA as a study of tonsillectomy in children under 2 years of age showed that children with DS made up 13% of the cohort and 25% of children with severe OSA (46). While the majority of children were reported to use nasal masks for LT-NIV, there appears to be a higher use of full face masks in children with DS as compared to other children. This may be secondary to craniofacial features such as flat nasal bridge, small nose, and hypoplasia of the maxilla (47, 48) that result in poor fit of commercially available NIV masks. A mismatch between the craniofacial features of children with DS and masks may also account for higher rates of leak and higher residual AHI. As nasal masks are generally recommended as the starting point for LT-NIV, (49) poor fit may contribute to difficulties with adherence and a need for additional support around initiation of LT-NIV, longer time to establish NIV use, and higher rates of inability to establish NIV use. Children with DS may be a group where custom masks would be beneficial. While there may be some unique features of LT-NIV use in children with DS, overall, the available data supports a similar approach to other children when considering and supporting LT-NIV use.

One question that cannot be addressed by the results of this systematic review is the best initial therapy for OSA in children with DS. Tonsillectomy and/or adenotonsillectomy are the recommended first line treatment for OSA in children if there is no contraindication to surgery (50–52). While there is a high rate of residual OSA after surgery in children with DS, 20-72% of children with DS have resolution of OSA after surgery (5–7). The one study in this systematic review that compared NIV and surgery used different criteria for initiation of therapy such that baseline characteristics of those undergoing surgery differ from those started on NIV (27). The results do suggest that NIV may be more effective at improving oxygen deficits in those with more severe OSA (minimal SpO2, % time with SpO2 <90%, oxygen desaturation index) as compared to surgery. Factors associated with residual OSA post-operatively in children with DS may include a smaller volume of the upper airway in the regions below the tonsils (53), smaller minimum airway area, and higher BMI (54). Virtual modeling of airway responses to surgery may help identify those children with DS who will benefit from surgical approaches to OSA (55). While randomized studies of the treatment of OSA in DS would be the ideal method to identify factors that predict treatment success, such studies may not be feasible given parental preferences for a treatment that may lead to cure over one that requires on-going treatment. It is, however, important to note that there is the potential for resolution of OSA, likely as a result of growth of the airway, such that LT-NIV can be ceased. This means that reassessment of the need for NIV is needed as is further work to identify factors associated with improvement or resolution of OSA in children with DS.

### Limitations of the Included Studies

The most prominent limitation of this systematic review is the serious risk of bias of the individual studies, which were mainly retrospective and observational. Risk of bias is serious overall due to baseline confounding variables, differences in type of support included in the definition of NIV (e.g., BPAP alone vs. CPAP/BPAP modes), limited consideration of adherence to NIV and subsequent impact on outcomes, loss of participants to follow-up, and selective reporting of results. As such, while this review provides a summary of current evidence, the serious risk of bias limits the ability to provide strong recommendations. In addition to limited outcomes data and comparisons of LT-NIV in children with and without DS, gaps in knowledge relevant to LT-NIV use in DS include the impact of craniofacial differences in DS on NIV use, the impact of LT-NIV use on facial growth, and an understanding of how LT-NIV use impacts quality of life for both children with DS and their caregivers.

### Limitations of the Review

The preceding scoping review did not include articles from all possible languages; this led to exclusion of 8% of articles which may have potentially included articles on children with DS. Furthermore, the search strategy was limited to children and may have excluded relevant studies on young adults where the mean age of subjects exceeded the set maximum of 18 years for this review. OSA risk in DS does not stop in adulthood (56, 57), therefore, conclusions of this review may not be applicable to young adults with DS. The heterogeneity of the subject groups and limited outcome data precluded meta-analysis.

## Conclusion

Children with DS make up a sizeable group of the children using LT-NIV. Despite this, there is limited data on the use and outcomes of this technology in children with DS. What is clear is that LT-NIV can be an effective and well-tolerated treatment option in many children with DS and that consideration for the use of this therapy should not differ for children with and without DS.

## Data Availability Statement

The original contributions presented in the study are included in the article/Supplementary Material, further inquiries can be directed to the corresponding author.

## Author Contributions

SH conceptualized and designed the study, designed the data collection instruments, collected the data, summarized the data, drafted the initial manuscript, reviewed, and revised the manuscript. MC-C and TA screened the manuscripts, reviewed, and revised the manuscript. MS provided methodological support including overseeing the search strategy, reviewed, and revised the manuscript. ML conceptualized the study, reviewed, and revised the manuscript. JM oversaw the project, conceptualized and designed the study, screened the manuscripts, reviewed, and revised the manuscript. All authors approved the final manuscript as submitted and agree to be accountable for all aspects of the work.

## Funding

This work was supported by the Alberta Strategy for Patient-Oriented Research (SPOR) Support Unit Knowledge Translation Platform, which was funded by Alberta Innovates and the Canadian Institutes of Health Research, and the Women and Children's Health Research Institute.

## Conflict of Interest

The authors declare that the research was conducted in the absence of any commercial or financial relationships that could be construed as a potential conflict of interest.

## Publisher's Note

All claims expressed in this article are solely those of the authors and do not necessarily represent those of their affiliated organizations, or those of the publisher, the editors and the reviewers. Any product that may be evaluated in this article, or claim that may be made by its manufacturer, is not guaranteed or endorsed by the publisher.
