# Supplementary material for: Long-Term Non-invasive Ventilation in Children With Down Syndrome: A Systematic Review
Source: Front Pediatr. 2022 May 23;10:886727. doi: 10.3389/fped.2022.886727 (PMC9168004; doi:10.3389/fped.2022.886727)
Supplement: Supplementary file 1 [file Table_1.DOCX]

Table 1; online only: Search strategy used in the Ovid Medline database for the scoping review to identify literature on the use of long-term non-invasive ventilation in children.

| Ovid MEDLINE(R) In-Process & Other Non-Indexed Citations & Ovid Medline(R): 1946 to November Week 1 2014 | |
| --- | --- |
| Original Search Date: 17 November 2014 | |
| Update Search Dates: 29 April 2016, 12 July 2017, 23 October 2019, 25 March 2021 | |
| 1. Continuous Positive Airway Pressure/  2. Noninvasive Ventilation/  3. Intermittent Positive-Pressure Breathing/  4. Ventilators, Negative-Pressure/  5. AVAPS.tw.  6. ((auto* or adaptive) adj2 (servoventilation or ventilation)).tw.  7. AutoSet*.tw.  8. ((bi level or bilevel) adj2 (airway* or air way* or assist* or breath* or positive pressure* or respirat* or ventilat* or support* or therap*)).tw.  9. BIPAP*.tw.  10. BPAP*.tw.  11. c flex.tw.  12. CNEP.tw.  13. (continuous negative adj2 pressure).tw.  14. (continuous positive airway* or continuous positive air way*).tw.  15. (continuous positive adj2 pressure).tw.  16. CPAP*.tw.  17. ((domicil* or home*) adj5 ventilat*).tw.  18. intermittent positive pressure breathing.tw.  19. IPPB*.tw.  20. ((long term or longterm) adj5 ventilat*).tw.  21. ((nasal* or mask*) adj2 (positive adj2 pressure)).tw.  22. ((nasal* or mask*) adj2 ventilat*).tw.  23. nCPAP*.tw.  24. ((negative pressure) adj2 (respirat* or ventilat*)).tw.  25. ((night* or nocturnal* or sleep*) adj5 ventilat*).tw.  26. NIPPV*.tw.  27. ((noninvasive adj5 ventilat*) or (non invasive adj5 ventilat*)).tw.  28. (noninvasive respiratory support* or non invasive respiratory support*).tw.  29. NPPV*.tw.  30. (positive pressure adj2 respirat*).tw.  31. REMstar*.tw.  32. (tank adj (respirat* or ventilat*)).tw.  33. VPAP*.tw.  34. or/1-33  35. Hypoventilation/pc, rh, th [Prevention & Control, Rehabilitation, Therapy]  36. Interactive Ventilatory Support/  37. Intermittent Positive-Pressure Ventilation/  38. Positive-Pressure Respiration/  39. Respiration, Artificial/  40. Respiratory Insufficiency/pc, rh, th [Prevention & Control, Rehabilitation, Therapy] | 41. exp Sleep Apnea Syndromes/ pc, rh, th [Prevention & Control, Rehabilitation, Therapy]  42. Ventilators, Mechanical/  43. ((airway* or air way* or breath* or inspirat* or respirat* or ventilat*) and (positive adj2 pressure)).tw.  44. intermittent positive pressure.tw.  45. IPPV*.tw.  46. (mechanical adj (respirat* or ventilat*)).tw.  47. (positive adj2 pressure adj (assist* or support* or therap*)).tw.  48. positive airway pressure.tw.  49. pulmonary ventilator*.tw.  50. respiratory support*.tw.  51. or/35-50  52. (noninvasive or non invasive or spontaneous*).mp.  53. 51 and 52  54. 34 or 53  55. exp Adolescent/  56. exp Child/  57. exp Infant/  58. exp Minors/  59. exp Pediatrics/  60. exp Puberty/  61. exp Schools/  62. adoles*.mp.  63. (baby* or babies or infant* or infancy or neonat* or newborn* or postmatur* or prematur* or preterm*).mp.  64. (boy* or girl* or teen*).mp.  65. (child* or kid or kids or preschool* or school age* or schoolchild* or toddler*).mp.  66. (elementary school* or high school* or highschool* or kindergar* or nursery school* or primary school* or secondary school*).mp.  67. minors*.mp.  68. (paediatric* or peadiatric* or pediatric*).mp.  69. (prepubescen* or pubescen* or pubert*).mp.  70. or/55-69  71. 54 and 70  72. (case reports or comment or editorial or letter).pt.  73. 71 not 72  74. exp animals/ not humans.sh.  75. 73 not 74  76. limit 75 to yr="1990 -Current"  77. remove duplicates from 76 |
